# The insights into the evolutionary history of *Translucidithyrium*: based on a newly-discovered species

**DOI:** 10.3897/mycokeys.75.58628

**Published:** 2020-12-17

**Authors:** Xinhao Li, Hai-Xia Wu, Jinchen Li, Hang Chen, Wei Wang

**Affiliations:** 1 International Fungal Research and Development Centre, The Research Institute of Resource Insects, Chinese Academy of Forestry, Kunming 650224, China The Research Institute of Resource Insects, Chinese Academy of Forestry Kunming China

**Keywords:** Divergence time, morphological characteristics, new species, Phaeothecoidiellaceae, phylogeny, speciation, taxonomy

## Abstract

During the field studies, a *Translucidithyrium*-like taxon was collected in Xishuangbanna of Yunnan Province, during an investigation into the diversity of microfungi in the southwest of China. Morphological observations and phylogenetic analysis of combined LSU and ITS sequences revealed that the new taxon is a member of the genus *Translucidithyrium* and it is distinct from other species. Therefore, *Translucidithyriumchinense***sp. nov.** is introduced here. The Maximum Clade Credibility (MCC) tree from LSU rDNA of *Translucidithyrium* and related species indicated the divergence time of existing and new species of *Translucidithyrium* was crown age at 16 (4–33) Mya. Combining the estimated divergence time, paleoecology and plate tectonic movements with the corresponding geological time scale, we proposed a hypothesis that the speciation (estimated divergence time) of *T.chinense* was earlier than *T.thailandicum*. Our findings provided new insights into the species of *Translucidithyrium* about ecological adaptation and speciation in two separate areas.

## Introduction

The sooty blotch and flyspeck fungi are widespread species and commonly occur on the surface of leaves, stems and fruits in tropical and subtropical zones (Yang et al. 2010; Gleason et al. 2011; Hongsanan et al. 2017; Zeng et al. 2018). Although these fungi do not directly harm host plants, they may affect the economic value of fruit sales ability and reduce photosynthesis in plants (Gleason et al. 2011). Sooty blotch fungi can form dark mycelial mats, whereas flyspeck fungi lack mycelial mats, form shiny and small, black spots (Batzer et al. 2005; Yang et al. 2010; Gleason et al. 2011; Zhang et al. 2015; Singtripop et al. 2016; Hongsanan et al. 2017). However, these fungi are poorly known, because of the difficulty in obtaining the strain which grows slowly (Yang et al. 2010; Hongsanan et al. 2017; Zeng et al. 2018).

Phaeothecoidiellaceae K.D. Hyde & Hongsanan was introduced by Hongsanan et al. (2017) and accommodated three genera *Chaetothyrina*, *Houjia* and *Phaeothecoidiella* in the order Capnodiales. Currently, it includes eight genera: *Chaetothyrina*, *Exopassalora*, *Houjia*, *Nowamyces*, *Phaeothecoidiella*, *Rivilata*, *Sporidesmajora* and *Translucidithyrium* (Hongsanan et al. 2020). Members of Phaeothecoidiellaceae are related to sooty blotch and flyspeck fungi and characterised by thyriothecia with setae, bitunicate asci and 1-septate ascospores (Singtripop et al. 2016; Hongsanan et al. 2017; Zeng et al. 2019; Hongsanan et al. 2020). *Chaetothyrina* is morphologically similar to the family Micropeltidaceae (Reynolds and Gilbert 2005), but is distinguishable by its brown upper wall of ascomata (Wu et al. 2019; Zeng et al. 2019). The genus *Rivilata* is placed in this family on the basis of morphological characters by Doilom et al. (2018). The *Nowamyces* was introduced as a new genus in the new family Nowamycetaceae by Crous et al. (2019) and Hongsanan et al. (2020) placed this genus into Phaeothecoidiellaceae by phylogenetic analysis. Hongsanan et al. (2020) listed *Houjia*, *Exopassalora*, *Sporidesmajora* and *Phaeothecoidiella* as asexual genera in Phaeothecoidiellaceae.

*Translucidithyrium* X.Y. Zeng & K.D. Hyde (2018) was introduced as a monotypic genus in Phaeothecoidiellaceae, which is represented by *T.thailandicum* X.Y. Zeng & K.D. Hyde (2018). It was characterised by epiphytes on the reverse of living leaves, semi-transparent ascomata, globose to subglobose asci and fusiform ascospores with verrucose and appendages. Ascospores germinated on MEA (Malt Extract Agar Medium) within 24 h. The colonies slowly grow on media, white to grey, circular and villiform (Zeng et al. 2018).

Liu et al. (2017) used the molecular clock approach to estimate the divergence time of the order Capnodiales crown age at 151–283 Mya (million years ago). Zeng et al. (2019) estimated the divergence time of the family Phaeothecoidiellaceae crown age at 40–60 Mya. The molecular clock approach for estimating divergence time might be used to predict speciation, historical climate change or other environmental events (Hélène and Arne 2014; Louca and Pennell 2020).

In this study, we collected an extraordinary new species of *Translucidithyrium* in Xishuangbanna, Yunnan Province, China. We described the morphological characteristics and built a phylogenetic tree to determine the classification of the new taxon. We compared and analysed the estimated divergence time of *Translucidithyrium* with the environmental changes around the corresponding time range to propose the evolutional history hypothesis of *Translucidithyrium* distributed in two different regions (China and Thailand).

## Methods

### Morphological

Fresh living leaves with olivaceous dots were collected at Xishuangbanna, China 21°55'51"N, 101°15'08"E, 540 m alt.) and delivered to the laboratory for observation. According to Wu et al. (2014), the collected samples were processed and examined by microscopes: the photos of ascomata were taken by using a compound stereomicroscope (KEYENCE CORPORATION V.1.10 with camera VH-Z20R). Hand sections were made under a stereomicroscope (OLYMPUS SZ61) and mounted in water and blue cotton and photomicrographs of fungal structures were taken with a compound microscope (Nikon ECLIPSE 80i). The single spore isolation was implemented by the methods of Choi et al. (1999) and Chomnunti et al. (2014). Germinated spores were individually transferred to PDA (Potato Dextrose Agar Medium) and incubated at 26 °C for 48 h. Colony characteristics were observed and measured after 4 weeks at 26 °C. Images used for figures were processed with Adobe Photoshop CC v. 2015.5.0 software (Adobe Systems, USA). The holotype was deposited at the herbarium of IFRD (International Fungal Research & Development Centre; Research Institute of Resource Insects, Kunming), reference number IFRD 9208. The ex-type strain was deposited at IFRDCC, reference number IFRDCC 3000.

### DNA isolation, amplification and sequencing

According to the manufacturer’s instructions, genomic DNA was extracted from mycelium growing on PDA at room temperature by using the Forensic DNA Kit (OMEGA, USA). The primer pair LR0R and LR5 was used to amplify the large subunit (LSU) rDNA (Vilgalys and Hester 1990). The primer pair ITS5 and ITS4 was used to amplify the internal transcribed spacer (ITS) rDNA (White et al. 1990). The primer pair NS1 and NS4 was used to amplify the partial small subunit (SSU) rDNA (White et al. 1990). The PCR reactions were in accordance with instructions from Golden Mix, Beijing TsingKe Biotech Co. Ltd, Beijing, China: initial denaturation at 98 °C for 2 min, then 30 cycles of 98 °C denaturation for 10 s, 56 °C annealing for 10 s and 72 °C extension for 10 s (ITS and SSU) or 20 s (LSU) and a final extension at 72 °C for 1 min. All PCR products were sequenced by Biomed (Beijing, China).

### Sequences alignments and phylogenetic analysis

BioEdit version 7.0.5.3 (Hall 1999) was used to re-assemble sequences generated from forward and reverse primers for obtaining the integrated sequences. Sequences were downloaded from GenBank using data from the publications of Zeng et al. (2018), Crous et al. (2019), Hongsanan et al. (2020) and Renard et al. (2020) and aligned using BioEdit version 7.0.5.3 (Hall 1999): in addition, sequences were adjusted manually.

Maximum Likelihood (ML) analysis was conducted by using RAxMLGUI v.1.0 (Silvestro and Michalak 2012). Aligned sequences were input into the software and *Dothideasambuci* was selected as the outgroup taxon. One thousand non-parametric bootstrap iterations were employed with the “ML + rapid bootstrap” tools and “GTRGAMMA” arithmetic.

For Bayesian analysis, MrModeltest 2.3 (Nylander 2004) was used to estimate the best-fitting model for the combined LSU and ITS genes. Posterior probabilities (Rannala and Yang 1996; Zhaxybayeva and Gogarten 2002) were determined by Markov Chain Monte Carlo (MCMC) sampling in MrBayes v.3.2 (Ronquist and Huelsenbeck 2003). Six simultaneous Markov chains were run for 2,000,000 generations; trees were printed every 1,000 generations; trees were sampled every 100 generations. The ﬁrst 5,000 trees submitted to the burn-in phase and were discarded; the remaining trees were used for calculating posterior probabilities in the majority rule consensus tree (Cai et al. 2006, 2008; Liu et al. 2012).

### Fossil calibrations and divergence time estimations

The fossil *Protographumluttrellii* (Renard et al. 2020) was used to calibrate the divergence time of Asterotexiales and Aulographaceae (normal distribution, mean = 119.0, SD = 3.7). The secondary calibration from the family Phaeothecoidiellaceae with a crown age of 58 Mya (normal distribution, mean = 50.0, SD = 6.1) was used (Zeng et al. 2019). The additional secondary calibration of Capnodiales was used, based on the result from Liu et al. (2017) (normal distribution, mean = 217.0, SD = 40.0).

Divergence time analysis was carried out using BEAST v1.8.4 (Drummond et al. 2012). Aligned LSU sequence data were loaded into the BEAUti v1.10.4 for generating an XML ﬁle. An uncorrelated relaxed clock model (Drummond et al. 2006) with a lognormal distribution of rates was used for the analysis. We used a Yule Process tree prior (Yule 1925; Gernhard 2008), which assumes a constant speciation rate per lineage and a randomly-generated starting tree. The length of chain was set as 50 million generations and sampling parameters were set at every 5,000 generations in MCMC. Subsequent divergence time analysis was carried out using BEAST v.1.10.4 (Drummond et al. 2012). Tracer v.1.7.1 was used to check the effective sample sizes (ESS) and acceptable values were higher than 200. The .log ﬁles and .tree ﬁles generated by BEAST were combined in LogCombiner v1.10.4 after removing a proportion of states as burn-in. The Maximum Clade Credibility (MCC) tree was given by obtained data and was estimated in TreeAnnotator v.1.10.4 (Liu et al. 2017; Zeng et al. 2019, 2020; Renard et al. 2020).

The phylogenetic tree and MCC tree were visualized in FigTree v.1.4.3 (Rambaut 2012) and Adobe Illustrator CS6 v. 16.0.0 (Adobe Systems, USA).

## Results

### Phylogenetic study

**Figure 1.**
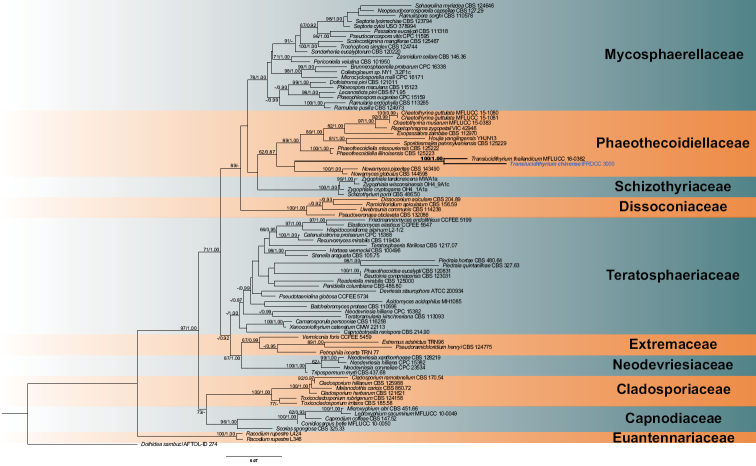
The topology shows family relationships of Capnodiales, based on combined LSU and ITS dataset analysis. Bootstrap values of Maximum Likelihood higher than 60% are shown on the left, while values of Bayesian posterior probabilities above 80% are shown on the right. New species is given in bold. Clades of the key species or family are given in bold. The tree is rooted with *Dothideasambuci* (Dothideaceae, Dothideales).

The dataset of combined LSU and ITS sequences comprised 1350 characters after alignment. Bayesian Inference, in total, generated 20,001 trees and the average standard deviation of split frequencies reached 0.0096. A total of 15,001 trees were finally used to calculate posterior probabilities. Phylogenetic analysis showed that the new collection clusters with *T.thailandicum* with 100% Maximum Likelihood bootstrap support and 1.00 posterior probabilities (Fig. 1).

**Table 1.** Selected taxa in this study with their corresponding GenBank accession numbers. The newly-generated sequences are shown in bold.

No.	Species	Vouncher /strain no.	LSU	ITS
1	*Acidomycesacidophilus*	MH1085	JQ172741	JQ172741
2	*Asterinaphenacis*	TH 589	GU586217	–
3	*Asterotexiaceae* sp.	VUL.535	MG844162	–
4	*Aulographum* sp.	VUL.457	MG844158	–
5	*Batcheloromycesproteae*	CBS 110696	JF746163	JF746163
6	*Baudoiniacompniacensis*	CBS 123031	GQ852580	–
7	*Brunneosphaerellaprotearum*	CPC 16338	GU214397	GU214626
8	*Buelliellaminimula*	Lendemer 42237(NY)	KX244961	–
9	*Camarosporulapersooniae*	CBS 116258	JF770461	JF770449
10	*Capnobotryellarenispora*	CBS 214.90	GU214398	AY220612
11	*Capnodiumcoffeae*	CBS 147.52	GU214400	DQ491515
12	*Catenulostromaprotearum*	CPC 15368	GU214402	GU214628
13	*Chaetothyrinaguttulata*	MFLUCC15–1080	KU358917	KX372277
14	*Chaetothyrinaguttulata*	MFLUCC15–1081	KU358914	KX372276
15	*Chaetothyrinamusarum*	MFLUCC 15–0383	KU710171	–
16	*Cladosporiumherbarum*	CBS 121621	KJ564331	EF679363
17	*Cladosporiumhillianum*	CBS 125988	KJ564334	HM148097
18	*Cladosporiumramotenellum*	CBS 170.54	DQ678057	AY213640
19	*Colletogloeum* sp.	NY1_3.2F1c	FJ031986	FJ425193
20	Conidiocarpus (Phragmocapnias) betle	MFLUCC 10–0050	JN832605	–
21	*Devriesiastaurophora*	ATCC 200934	KF901963	AF393723
22	*Dissoconiumaciculare*	CBS 204.89	GU214419	AY725520
23	*Dothideasambuci*	AFTOL-ID 274	AY544681	DQ491505
24	*Dothistromapini*	CBS 121011	JX901821	JX901734
25	*Elasticomyceselasticus*	CCFEE 5547	KF309991	–
26	*Exopassalorazambiae*	YHJN13	GQ433631	GQ433628
27	*Extremusadstrictus*	TRN96	KF310022	–
28	*Friedmanniomycesendolithicus*	CCFEE 5199	KF310007	JN885547
29	*Hispidoconidiomaalpinum*	L2–1/2	FJ997286	FJ997285
30	*Hortaeawerneckii*	CBS 100496	GU301817	AY128703
31	*Houjiayanglingensis*	YHJN13	GQ433631	GQ433628
32	*Lecanostictapini*	CBS 871.95	GQ852598	–
33	*Lembosiaalbersii*	MFLUCC 13–0377	KM386982	–
34	*Lembosina* sp.	VUL.644	MG844165	–
35	*Leptoxyphiumcacuminum*	MFLUCC 10–0049	JN832602	–
36	*Melanodothiscaricis*	CBS 860.72	GU214431	GU214638
37	*Microcyclosporellamali*	CPC 16171	GU570545	GU570528
38	*Microxyphiumcitri*	CBS 451.66	KF902094	–
39	*Morenoinacalamicola*	MFLUCC 14–1162	NG059779	NR154210
40	*Mycosphaerellapneumatophorae*	AFTOL-ID 762	KJ176856	–
41	*Neodevriesiacoryneliae*	CPC 23534	KJ869211	KJ869154
42	*Neodevriesiahilliana*	CPC 15382	GU214414	GU214633
43	*Neodevriesiaxanthorrhoeae*	CBS 128219	HQ599606	HQ599605
44	*Neopseudocercosporellacapsellae*	CBS 127.29	KF251830	KF251326
45	*Nowamycesglobulus*	CBS 144598	MN162196	MN161935
46	*Nowamycespiperitae*	CBS 143490	MN162200	MN161944
47	*Parapenidiellatasmaniensis*	CBS 124991	KF901844	KF901522
48	*Passaloraeucalypti*	CBS 111318	KF901938	KF901613
49	*Penidiellacolumbiana*	CBS 486.80	EU019274	KF901630
50	*Periconiellavelutina*	CBS 101950	EU041840	EU041783
51	*Petrophilaincerta*	TRN 77	GU323963	–
52	*Phaeophleosporaeugeniae*	CPC 15159	KF902095	KF901742
53	*Phaeothecoideaeucalypti*	CBS 120831	KF901848	KF901526
54	*Phaeothecoidiellaillinoisensis*	CBS 125223	GU117901	GU117897
55	*Phaeothecoidiellamissouriensis*	CBS 125222	AY598917	AY598878
56	*Phloeosporamaculans*	CBS 115123	GU214670	GU214670
57	*Piedraiahortae*	CBS 480.64	GU214466	GU214647
58	*Piedraiaquintanilhae*	CBS 327.63	GU214468	–
59	*Pseudocercosporavitis*	CPC 11595	GU214483	GU269829
60	*Pseudoramichloridiumhenryi*	CBS 124775	KF442561	KF442521
61	*Pseudotaeniolinaglobosa*	CCFEE 5734	KF310010	KF309976
62	*Pseudoveronaeaobclavata*	CBS 132086	JQ622102	–
63	*Racodiumrupestre*	L346	EU048583	GU067666
64	*Racodiumrupestre*	L424	EU048582	GU067669
65	*Ramichloridiumapiculatum*	CBS 156.59	EU041848	EU041791
66	*Ramulariaendophylla*	CBS 113265	AY490776	AY490763
67	*Ramulariapusilla*	CBS 124973	KP894141	KP894248
68	*Ramulisporasorghi*	CBS 110578	GQ852653	-
69	*Readeriellamirabilis*	CBS 125000	KF251836	KF251332
70	*Recurvomycesmirabilis*	CBS 119434	GU250372	FJ415477
71	*Repetophragmazygopetali*	VIC42946	KT732418	
72	*Schizothyriumpomi*	CBS 486.50	EF134948	EF134948
73	*Scolecostigminamangiferae*	CBS 125467	GU253877	GU269870
74	*Scoriasspongiosa*	CBS 325.33	GU214696	GU214696
75	*Septoriacytisi*	USO 378994	JF700954	JF700932
76	*Septorialysimachiae*	CBS 123794	KF251972	KF251468
77	*Sonderheniaeucalyptorum*	CBS 120220	KF901822	KF901505
78	*Sphaerulinamyriadea*	CBS 124646	JF770468	JF770455
79	*Sporidesmajirapennsylvaniensis*	CBS 125229	MH874965	MF951287
80	*Stenellaaraguata*	CBS 105.75	EU019250	EU019250
81	*Teratoramulariakirschneriana*	CBS 113093	GU214669	GU214669
82	*Teratosphaeriafibrillosa*	CBS 1217.07	GU323213	KF901728
83	*Toxicocladosporiumirritans*	CBS 185.58	EU040243	EU040243
84	*Toxicocladosporiumrubrigenum*	CBS 124158	FJ790305	FJ790287
85	***Translucidithyriumchinense***	**IFRDCC 3000**	**MT659404**	**MT659671**
86	*Translucidithyriumthailandicum*	MFLUCC 16–0362	MG993048	MG993045
87	*Tripospermummyrti*	CBS 437.68	GU323216	–
88	*Trochophorasimplex*	CBS 124744	GU253880	GU269872
89	*Uwebrauniacommunis*	CBS 114238	EU019267	AY725541
90	*Vermiconiaforis*	CCFEE 5459	GU250390	KF309981
91	*Xenoconiothyriumcatenatum*	CMW 22113	JN712570	JN712502
92	*Zasmidiumcellare*	CBS 146.36	EU041878	EU041821
93	*Zygophialacryptogama*	OH4_1A1a	FJ147157	FJ425208
94	*Zygophialatardicrescens*	MWA1a	EF164901	AY598856
95	*Zygophialawisconsinensis*	OH4_9A1c	FJ147158	FJ425209

### Taxonomy

#### Translucidithyrium chinense

Taxon classificationFungiCapnodialesPhaeothecoidiellaceae

H. X. Wu & X. H. Li sp. nov.

5AEB5C48-DFE8-5556-951F-06AEE74F84E0

Index Fungorum number: IF 557843

Facesoffungi number: FoF 09429

Figures 2 , 3

##### Etymology.

Refer to the location of species, China.

##### Holotype.

IFRD9208

##### Description.

**Figure 2.**
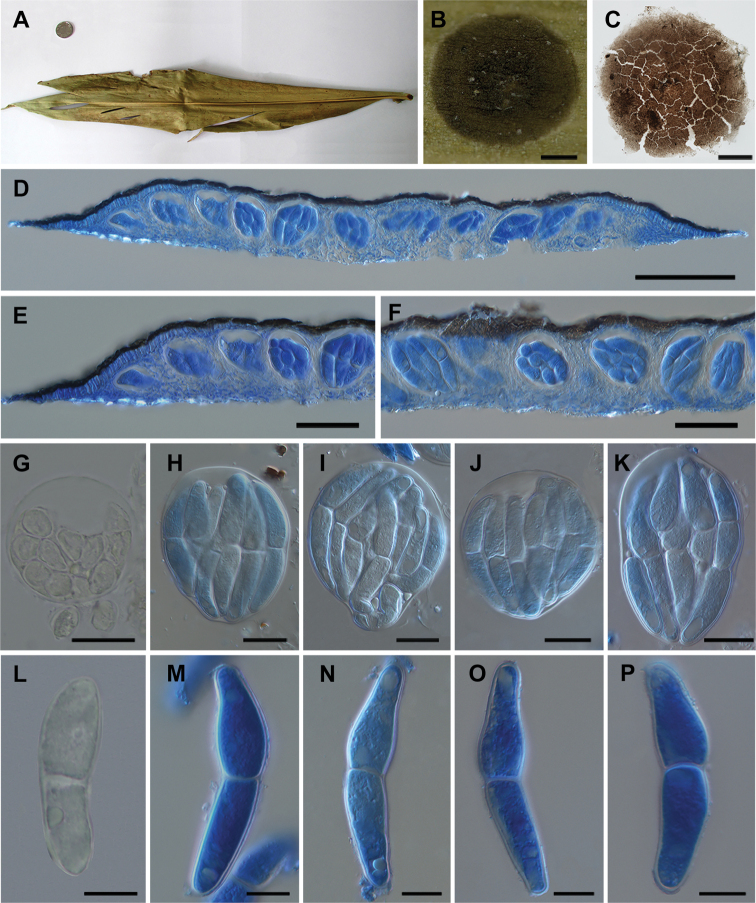
*Translucidithyriumchinense* (IFRD 9208, holotype) **A** plant leaves **B** acscoma on leaves surface **C** squash of ascoma at 20 times amplification **D** cross section of ascoma in blue cotton at 20 times amplification **E, F** cross section of ascoma in blue cotton at 40 times amplification **G** asci at 100 times amplification **H–K** asci in blue cotton at 100 times amplification **L** ascospore at 100 times amplification **M–P** ascospore in blue cotton at 100 times amplification. Scale bars: 200 µm (**B**); 100 µm (**C, D**); 50 µm (**E, F**); 20 µm (**G–K**); 10 µm (**L–P**). We slightly adjusted the contrast, saturation and hue of images and removed the contaminants around main object in images in PS software without obscuration, erasure or distortion of any information existing in the original document.

*Epiphytic* on living leaves, ascomata with papillate. *Superficialhyphae* absent. **Sexual morph**: *Ascomata* solitary or scattered, 480–870 μm diam. (x̄ = 741 μm, n = 6), 65–82 µm high (x̄ = 72 μm, n = 8), olivaceous to brown, slightly semi-transparent under highlighted background, circular to suborbicular, with slightly prominent papilla, membranous, without ostiole (Fig. 2A–C). *Peridium* 8.3–10 μm thick, (x̄ = 9 μm, n = 11), composed of irregular, meandering, interwoven arranged cells, two layers: from brown to hyaline, outer layer composed of closely-arranged cells, brown; inner layer composed of hyaline, oblong, subdense arranged cells, poorly developed at the base (Fig. 2D–F). *Asci* evenly distributed and parallel arranged in hamathecium (Fig. 2D–F), 65–90 × 51–81 μm (x̄ = 77 × 60 μm, n = 10), 8-spored, bitunicate, hyaline, with an ocular chamber, ovoid at immature state, globose to subglobose at mature state, lacking pedicel, paraphyses absent (Fig. 2G–K). *Ascospores* 41–65 × 10–13 μm (x̄ = 50 × 11 μm, n = 20), irregularly overlapping, hyaline, ovoid at young state, fusiform with both ends tapered at mature state, 1-septate, constricted at the septum, upper cell a little larger than lower, with guttules at both ends, verrucose (Fig. 2L–P). **Asexual morph**: Undetermined.

##### Culture characteristics.

**Figure 3.**
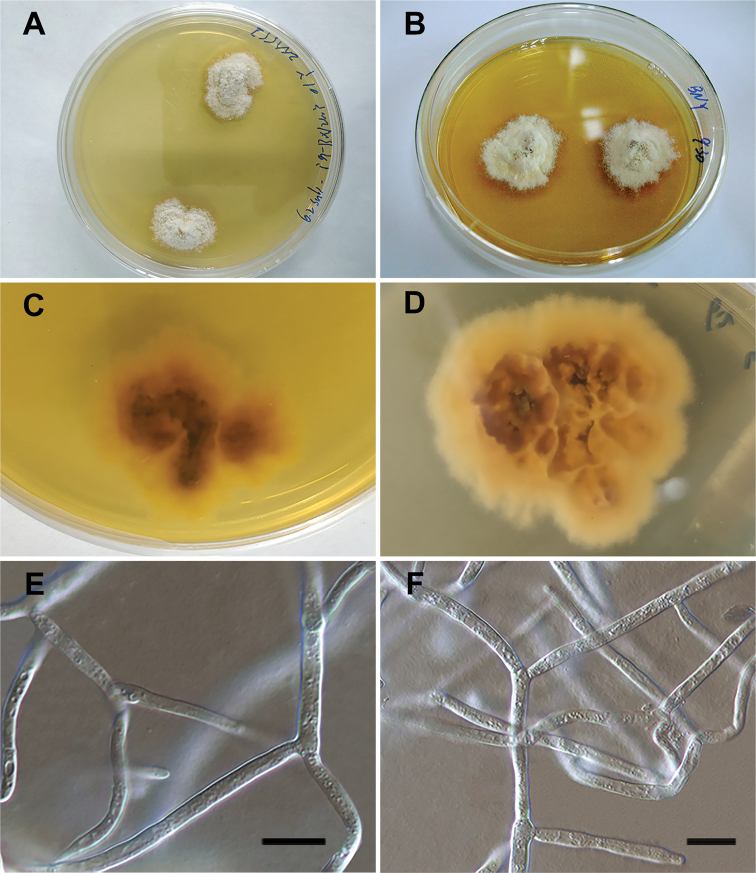
Culture of *Translucidithyriumchinense* (IFRDCC3000) **A, B** culture growing on the medium **C, D** the bottom of the medium with culture growing **E, F** the mycelium of culture at 100 times amplification. Scale bars: 10 µm (**E, F**).

Ascospores germinating on MEA at 36 h after spore-isolation, germinating on PDA at 48 h after spore-isolation. Colonies slow growing on MEA and PDA, irregular, villiform, convex, white on surface, yellow to brown at base. After a long period of growth, the pigments produced by culture discolour the medium, roots generate at the bottom (Fig. 3A–D). Culture hyphae hyaline, branched, constricted at the septum, 3 μm wide (Fig. 3E, F).

##### Material examined.

China, Yunnan Province, Xishuangbanna Dai Autonomous Prefecture, Xishuangbanna Botanical Garden; 21°55'51"N, 101°15'08"E, 540 m alt.; 21 Apr 2019; Haixia Wu and Xinhao Li leg; collected on living leaves of *Alpiniablepharocalyx* (IFRD 9208, holotype), ex-type living culture (IFRDCC 3000).

##### Notes.

This new species is morphologically similar to *Translucidithyriumthailandicum* in having semi-transparent and largish ascomata, globose asci and hyaline ascospores with 1-septate. However, *Translucidithyriumchinense* has a slightly papilla thyriothecium with weaker transmittance and ascospores with guttules at both ends, while *T.thailandicum* has a flattened thyriothecium with higher transmittance and ascospores with appendages at both ends; besides, the size of ascomata and asci of *T.chinense* are slightly larger than those of *T.thailandicum* (795 μm vs. 621 μm; 77 μm vs. 64 μm). The culture characteristics of both species are different: the culture of *T.chinense* grows more slowly, has roots inserting into medium and turn the bottom brown. Phylogenetically, *T.chinense* clusters with *T.thailandicum* as a distinct clade with high support (100% ML / 1.00 PP, Fig. 1).

##### Divergence times estimates.

The Maximum Clade Credibility (MCC) tree was similar to the major lineages in the Bayesian and ML trees. The crown age of *Translucidithyrium* showed 16 Mya (4–33), which was earlier than the divergence time of most genera in Phaeothecoidiellaceae. The estimated divergence time of Phaeothecoidiellaceae from Zeng et al. (2019) is 58 Mya, which corresponds to our results.

## Discussion

*Translucidithyriumthailandicum* was found in the north of Thailand (Zeng et al. 2018). *Translucidithyriumchinense* was found in the Xishuangbanna Region, southwest of China, which lies on the northern border of a rainforest with rich microfungal resources. The new species is characterised by brown to olivaceous ascomata and slightly semi-transparent, subglobose asci without pedicel and fusiform ascospores with verrucose and guttules (Fig. 2). *T.chinense* is introduced as a new species in *Translucidithyrium* by morphological and phylogenetic studies (Figs 1–3).

The ascomata of *Translucidithyrium* are different from related genera of Phaeothecoidiellaceae: *Nowamyces* has immersed ascomata, *Chaetothyrina* has ascomata with setae and *Rivilata* has subcuticular ascomata (Singtripop et al. 2016; Doilom et al. 2018; Zeng et al. 2018; Crous et al. 2019; Hongsanan et al. 2020). *Translucidithyrium* is similar to the family Schizothyriaceae in having semi-transparent ascomata, globose to subglobose asci and hyaline ascospores with guttules. Schizothyriaceae includes *Schizothyrium*, *Plochmopeltis*, *Hexagonella*, *Lecideopsella*, *Mycerema*, *Kerniomyces*, *Metathyriella*, *Myriangiella*, *Amazonotheca* and *Vonarxella* (Phookamsak et al. 2016; Wijayawardene et al. 2020). The morphology of *T.chinense* is most similar to *Lecideopsella* by having globose asci and 1-septate ascospores, but *Lecideopsella* has a short pedicel at the bottom of the asci (Phookamsak et al. 2016; Zeng et al. 2018). Phylogenetically, *Translucidithyrium* formed a long clade and clustered within the family Phaeothecoidiellaceae. It indicated the existing certain genetic distance amongst *Translucidithyrium*, Phaeothecoidiellaceae and Schizothyriaceae. Phaeothecoidiellaceae and Schizothyriaceae are poorly studied families (Batzer et al. 2008; Phookamsak et al. 2016; Singtripop et al. 2016; Hongsanan et al. 2017; Zeng et al. 2018). Therefore, more fresh specimens with molecular data are needed to confirm the classification of *Translucidithyrium*, Phaeothecoidiellaceae and Schizothyriaceae.

**Figure 4.**
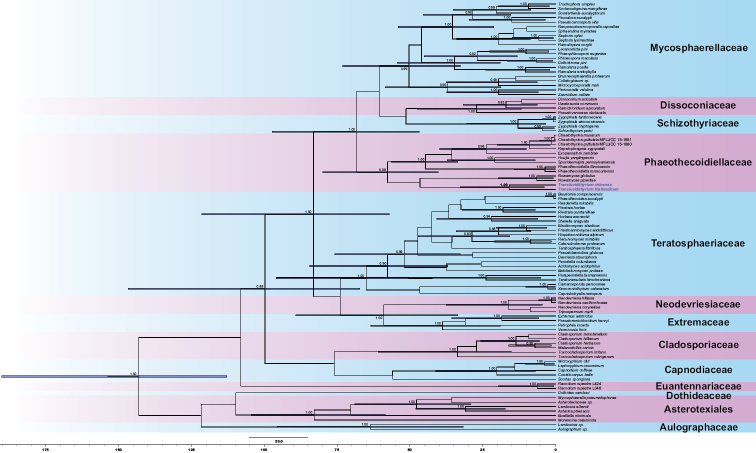
The MCC tree with divergence times estimates of Phaeothecoidiellaceae obtained from a Bayesian approach (BEAST). Numbers at nodes indicate posterior probabilities (pp) for node support; bars correspond to the 95% highest posterior density (HPD) intervals. The key species are given in blue.

Zuckerkandl and Pauling (1962) suggested that the number of differences amongst amino acids was proportional to species divergence time. We estimated the divergence time using BEAST analysis. The divergence time of *Translucidithyrium* crown age was estimated at 16 Mya (4–33), which was earlier than the crown ages of *Chaetothyrina* at 2 Mya (0–5), the crown ages of *Repetophragma* at 9 Mya (2–20), the crown ages of *Nowamyces* at 7 Mya (1–20) and the crown ages of *Phaeothecoidiella* at 4 Mya (0–14) within Phaeothecoidiellaceae (Fig. 4). The divergence time of *Translucidithyrium* is earlier than other genera in Phaeothecoidiellaceae. We estimate that the long divergence time should affect the genetic variation (Pauling 1964; Hall and Hallgrímsson 2008). Additionally, the evolutionary molecular clock approach confirmed the long clades of *Translucidithyrium* in the phylogenetic tree (Fig. 1).

Historical events amongst different biological groups could then be compared with the dates of plate tectonic movements and paleoecology, according to the corresponding geological time scale (Lomolino et al. 2006; Berbee and Taylor 2010). Through relevant studies on the Qinghai-Tibet Plateau, it was found that the time of intense tectonic uplift and denudation is concentrated in 60–35 Mya, 25–17 Mya, 12–8 Mya and 5 Mya. Global cooling might have an impact on climate change in East Asia, especially at 15 Mya and 8 Mya (Lu et al. 2010). Rising plateaus and global cooling were drying up Asia (Liu 2000; Garzione et al. 2015). The time of the Qinghai-Tibet Plateau uplift and global cooling corresponded to the interval of the species in *Translucidithyrium* divergence time. We predict that the speciation of *T.chinense* was earlier than the speciation of *T.thailandicum*, as the divergence of *Translucidithyrium* was related to the Qinghai-Tibet Plateau uplift and global cooling. According to the evolution history of *Translucidithyrium*, it could be speculated that the speciation of *T.chinense* was earlier than *T.thailandicum*. With the climate becoming colder and with increased drought, *T.chinense* migrated from China to Thailand gradually to find a suitable area, then *T.thailandicum* formed. Due to the end of global cooling, the distribution pattern of *Translucidithyrium* in two different countries formed. Increasing fresh collections and application of new methodologies may result in modified conclusions.

## Supplementary Material

XML Treatment for Translucidithyrium chinense
